# Supplementary material for: Heat adaptation of phage T7 under an extended genetic code
Source: Virus Evol. 2021 Dec 1;7(2):veab100. doi: 10.1093/ve/veab100 (PMC8923235; doi:10.1093/ve/veab100)
Supplement: veab100_Supp [file veab100_supp.zip › Sequencing_Analysis.zip!/Sequencing_Analysis/A1/evidence/JC_66.html]

BRESEQ :: Evidence


| New junction evidence | | | | | | | | | | | |
| --- | --- | --- | --- | --- | --- | --- | --- | --- | --- | --- | --- |
|  | | seq id | position | reads (cov) | reads (cov) | score | skew | freq | annotation | gene | product |
| \* | ? | NC\_001604.1 | = 36882 | 3450 (1.120) | 16 (0.010) | 15/582 | 37.4 | 0.48% | intergenic (–/–) | *–/–* | –/– |
|  | ? | NC\_001604.1 | 37265 = | 3289 (1.100) |  |  |  |  | intergenic (–/–) | *–/–* | –/– |
| Rejected: Coverage evenness skew score above cutoff. | | | | | | | | | | | |
| Rejected: Frequency below/above cutoff threshold. | | | | | | | | | | | |

Only 100 of 339 total aligned reads displayed.  

`ATTACATTCTTAGAGATGTTGGACACTGCGATGGCTCAGCGTATGCTTGCGGACCTTTCGGACCATGAGCGTCGCTCTCCGCAACTCTATAATGCTATTAACAAACTGTTAGACCGCCACAAGTTCCAGATTGGTAAGTTGCAGCCGGATGTTCACATCTTAGGTGGCCTTGCTGGTGCTCTTGAAGAGTACAAAGAGAAAGTCGGTGATAACGGTCTTACGGATGATGATATTTACACATTACAGTGATATACTCAAGGCCACTACAGATAGTGGTCTTTATGGATGTCATTGTCTATACGAGATGCT‑‑‑‑‑‑‑‑‑‑‑‑‑‑‑‑‑‑‑‑‑‑‑‑‑‑‑‑‑‑‑‑‑‑‑‑‑‑‑‑‑‑‑‑‑‑‑‑‑‑‑‑‑‑‑‑‑‑‑‑‑‑‑‑‑‑‑‑‑‑‑‑‑‑‑‑‑‑‑‑‑‑‑‑‑‑‑‑‑‑‑‑‑‑‑‑‑‑‑‑‑‑‑‑‑‑‑‑‑‑‑‑‑‑‑‑‑‑‑‑‑‑‑‑‑‑‑‑‑‑‑‑‑‑‑‑‑‑‑‑‑‑‑‑‑‑‑‑‑‑‑‑‑‑‑‑‑‑‑‑‑‑‑‑‑‑‑‑‑‑‑‑‑‑‑‑‑‑‑‑‑‑‑‑‑‑‑‑‑‑‑‑‑‑‑‑‑‑‑‑‑‑‑‑‑‑‑‑‑‑‑‑‑‑‑‑‑‑‑‑‑‑‑‑‑‑‑‑‑‑‑‑‑‑‑‑‑‑‑‑‑‑‑‑‑‑‑‑‑‑‑‑‑‑‑‑‑‑‑‑‑‑‑‑‑‑‑‑‑‑‑‑‑‑‑‑‑‑‑‑‑‑‑‑‑‑‑‑‑‑‑‑‑‑‑‑‑‑‑‑  >  NC_001604.1/36574‑36882`  
`‑‑‑‑‑‑‑‑‑‑‑‑‑‑‑‑‑‑‑‑‑‑‑‑‑‑‑‑‑‑‑‑‑‑‑‑‑‑‑‑‑‑‑‑‑‑‑‑‑‑‑‑‑‑‑‑‑‑‑‑‑‑‑‑‑‑‑‑‑‑‑‑‑‑‑‑‑‑‑‑‑‑‑‑‑‑‑‑‑‑‑‑‑‑‑‑‑‑‑‑‑‑‑‑‑‑‑‑‑‑‑‑‑‑‑‑‑‑‑‑‑‑‑‑‑‑‑‑‑‑‑‑‑‑‑‑‑‑‑‑‑‑‑‑‑‑‑‑‑‑‑‑‑‑‑‑‑‑‑‑‑‑‑‑‑‑‑‑‑‑‑‑‑‑‑‑‑‑‑‑‑‑‑‑‑‑‑‑‑‑‑‑‑‑‑‑‑‑‑‑‑‑‑‑‑‑‑‑‑‑‑‑‑‑‑‑‑‑‑‑‑‑‑‑‑‑‑‑‑‑‑‑‑‑‑‑‑‑‑‑‑‑‑‑‑‑‑‑‑‑‑‑‑‑‑‑‑‑‑‑‑‑‑‑‑‑‑‑‑‑‑‑‑‑‑‑‑‑‑‑‑‑‑‑‑‑‑‑‑‑‑‑‑‑‑‑‑‑‑‑cgagatgctAAACGTATTCTCGCAGTGACCCAGAAGGGTGACGCATGGATTCGTGCGTTACAGGATACTATTCGTGAACTGCAACGTAAGTAGGAAATCAAGTAAGGAGGCAATGTGTCTACTCAATCCAATCGTAATGCGCTCGTAGTGGCGCAACTGAAAGGAGACTTCGTGGCGTTCCTATTCGTCTTATGGAAGGCGCTAAACCTACCGGTGCCCACTAAGTGTCAGATTGACATGGCTAAGGTGCTGGCGAATGGAGACAACAAGAAGTTCATCTTACAGGCTTTCCGTGGTATCGGTAAGTCG  >  NC_001604.1/37265‑37564`  

                                                                                                                                                                                                                                                                                                                                                                                                                                                                                                                                                                                                                                   
`tTTACATTCTTAGAGCTGTTGTACACTGCGATGGCTCAGCGTATGCTTGCGGACCTTTCGGACCATGAGCGTGGCTCTCCGCAACTCTATAATGCTATTAACAAACTGTTAGACCGCCACAAGTTCCAGATTGG‑AAGTTGCAGCCGGATGTTCACATCTTAGGTGGCCTTGCTGGTGCTCTTGAAGAGTACAAAGAGAAAGTCGGTGATAACGGTCTTACGGATGATGATATTTACACATTACAGTGATATACTCAAGGCCACTACAGATAGTGGTCTTTATGGATGTCATTGTCTATACG                                                                                                                                                                                                                                                                                                                     <  1:259020/300‑1`  
`ATTACATTCTTAGAGATGTTGGACACTTCGATGGCTCAGCGTATGCTTGCGGACCTTTCGGACCATGAGCGTCGCTCTCCGCAACTCTATAATGCTATTAACAAACTGTTAGACCGCCACAAGTTCCAGATTGGTAAGTTGCAGCCGGATGTTCACATCTTAGGTGGCCTTACTGGTGCTCTTGAAGAGTACAAAGAGAAAGTCGGTGATAACGGTCTTACGGATGATTATATTTACACATTACAGTGATATACTCAAGGCCACTACAGATAGTGGTCTTTATGGATGTCATTGTCTATAC                                                                                                                                                                                                                                                                                                                      >  1:255009/1‑301`  
`ATTACATTCTTAGAGATGTTGGACACTGCGATGGCTCAGCGTATGTTTGCGGACCTTTCGGACCATGAGCGTCGCTCTCCGCAACTCTATAATGCTATTAACAAACTGTTAGACCGCCACAAGTTCCAGATTGGTAAGTTGCGGCCGGATGTTCACATCTTAGGTGGCCTTGCTGGTGCTCTTGAAGAGTACAAAGAGAAAGTCGGTGATAACGGGCTTACGGATGATGATATTTACACATTACAGTGATATACTCAAGGCAACTACAGATAGTGGTCTTTATGGATGTCATTGTCTATAC                                                                                                                                                                                                                                                                                                                      >  1:508795/1‑301`  
`ATTACATTCTTAGAGATGTTGGACACTGCGATGGCTCAGCGTATGCTTGCGGACCTTTCGGAGCATGAGCGTCGCTCTCCGCAACTCTATAATGCTATTAACAAACTGTTAGCCCGCCACAAGTTCCAGATTGGTAAGTTGCAGCCGGATGTTCACATCTTAGGTGGCCTTGCTGGTGCTCTTGCAGAGTACAAAGAGAAAGTCGGTGATAACGGTCGTACGGATGATGATAGTTACACATTACAGTGATATACTCAAGGCCACTACAGATAGTGGTCTTGATGGATATCATTGTATATAC                                                                                                                                                                                                                                                                                                                      >  1:260613/1‑301`  
`ATTACATTCTTAGAGATGTTGGACACTGCGATGGCTCAGCGTATGCTTGCGGACCTTTCGGACCATGAGCGTCGCTCTCCGCAACTCTATAATGCTATTAACAAACTGTTAGACCGCCACAAGTTCCAGATTGGTAAGTTGCAGCCGGATGTTCACATCTTAGGTGTCCTTGCTGGTGCTCTTGAAGAGTACAAAGAGAAAGTCGGTGATAACGGTCTTACGGATGTTGATATTTACACATTACAGTGATATACTCAAGGCCACTACAGATAGTGGTCTTTATGGATGTCATTGTCTATAC                                                                                                                                                                                                                                                                                                                      >  1:28572/1‑301`  
`ATTACATTCTTAGAGATGTTGGACACTGCGATGGCTCAGCGTATGCTTGCGGACCTTTCGGACCATGAGCGTCGCTCTCCGCAACTCTATAATGCTATTAACAAACTGTTAGACCGCCACAAGTTCCAGATTGGTAAGTTGCAGCCGGATGTTCACATCTTAGGTGGCCTTGCTGGTGCTCTTGAAGAGTACAAAGAGAAAGTCGGTGATAACGGTCTTACGGATGATGATATTTACACATTACAGTGATATACTCAAGGCCACTACAGATAGTGGTCTTTATGGATGTCATTGTCTATAC                                                                                                                                                                                                                                                                                                                      >  1:79239/1‑301`  
`ATTACATTCTTAGAGATGTTGGACACTGCGATGGCTCAGCGTATGCTTGCGGACCTTTCGGACCATGAGCGTCGCTCTCCGCAACTCTATAATGCTATTAACAAACTGTTAGACCGCCACAAGTTCCAGATTGGTAAGTTGCAGCCGGATGTTCACATCTTAGGTGGCCTTGCTGGTGCTCTTGAAGAGTACAAAGAGAAAGTCGGTGATAACGGTCTTACGGATGATGATATTTACACATTACAGTGATATACTCAAGGCCACTACAGATAGTGGTCTTTATGGATGTCATTGTCTATAC                                                                                                                                                                                                                                                                                                                      >  1:324915/1‑301`  
`ATTACATTCTTAGAGATGTTGGACACTGCGATGGCTCAGCGTATGCTTGCGGACCTTTCGGACCATGAGCGTCGCTCTCCGCAACTCTATAATGCTATTAACAAACTGTTAGACCGCCACAAGTTCCAGATTGGTAAGTTGCAGCCGGATGTTCACATCTTAGGTGGCCTTGCTGGTGCTCTTGAAGAGTACAAAGAGAAAGTCGGTGATAACGGTCTTACGGATGATGATATTTACACATTACAGTGATATACTCAAGGCCACTACAGATAGTGGTCTTGAGGGATGTCATTGTCTATAC                                                                                                                                                                                                                                                                                                                      >  1:492834/1‑301`  
`ATTACATTCTTAGAGATGTTGGACACTGCGATGGCTCAGCGTATGCTTGCGGACCTTTCGGACCATGAGCGTCGCTCTCCGCAACTCTATAATGCTATTAACAAACTGTTAGACCGCCACAAGTTCCAGATTGGTAAATTGCAGCCGGATGTTCACATCTTAGGTGGCCTTGCTGGTGCTCTTGAAGAGTACAAAGAGAAAGTCGGTGATAACGGTCTTACGGATGATGATATTTACACATTACAGTGATATACTCAAGGCCACTACAGATAGTGGTCTTTATGGATGTCATTGTCTATAC                                                                                                                                                                                                                                                                                                                      >  1:228202/1‑301`  
`ATTACATTCTTAGAGATGTTGGACACTGCGATGGCTCAGCGTATGCTTGCGGACCTTTCGGACAATGAGCGTCGCTCTCCGCAACTCTATAATGCTATTAACAAACTGTTAGACCGCCACAAGTTCCAGATTGGTAAGTTGCAGCCGGATGTTCACATCTTAGGTGGCCTTGCTGGTGCTCTTGAAGAGTACAAAGAGAAAGTCGGTGATAACGGTCTTACGGATGATGATATTTACACATTACAGTGATATACTCAAGGCCACTACAGATCGTGGTCTTTATGGATGTCATTGTATATAC                                                                                                                                                                                                                                                                                                                      >  1:338163/1‑301`  
`ATTACATTCTTAGAGATGTTGGACACTGCGATGGCTCAGCGTATGCTGGCGGACCTTTCGGACCATGAGCGTCGCTCTCCGCAACTCTATAATGCTATTAACAAACTGTTAGACCGCCACAAGTTCAAGATTGGTAAGTTGCAGACGGATGTTAACATCTTAGGTGGCCTTGCTGGTGCTCTTGAAGAGTACAAAGAGAAAGTCGGTGTTAACGGGCTTACGGATGATTATATTTAAACATTCCAGTGATATACTAAAGTCCACTACAGATAGTGGTCTTTATGGATGTCATTGTTGATAC                                                                                                                                                                                                                                                                                                                      >  1:284357/1‑301`  
`ATTACATTC‑TAGAGATGTTGGACACTGCGATGGCTCAGTGTATGCTTGCGGACCTTTCTGACCATGAGCGTCGCTCTCCGCAACTCTATAATGCTATTAACAAACGGTTAGACCGCCACAAGTTCCAGATTGGTAAGTTGCAGCCGGAGGTTCACATCTTAGGTGGCCTTGCTGGTGCTCTTGAAGAGTACAAAGAGAAAGTCGGGGATAACGGTCTTACGGATGATGATATTTACACATTACAGTGATATACTCAAGGCCACTACAGATAGTGGTCTTTATGGATGTCATTGTCTATACG                                                                                                                                                                                                                                                                                                                     <  1:417499/301‑1`  
 `tttaATTCTTAGAGATTGTGGACAAGGGGATGGTCCAGCGTATGCTTGCGGAACTTTCGGACCATGAGCGTCCCTCGCCTAAACTCTATAATGCTATTAACAAACGGTTAGACCGCAACAAGTTCCAGATTGGTAAGTTGCAGCCGGATGTTCACATCTTAGGTGGCCGTGCTGGTGCTCTTGAAGAGTACAAAGAGAAAGTCGGTGATAACGGTATTACGGATGATGATATTTACAAATTACAGTGATATACTCAAGGCCACTACAGATAGTGGTCTTTATGGATGTCATTGTCTATACG                                                                                                                                                                                                                                                                                                                     <  1:293648/297‑1`  
 `tgtgtataagagacagGTTGGACACTGCGATGGCTCAGCGTATGCTTGCGGACCTTTCGGACCATGAGCGTCGCTCTCCGCAACTCTATAATGCTATTAACAAACTGTTAGACCGCCACAAGTTCCAGATTGGTAAGTTGCAGCCGGATGTTCACATCTTAGGTGGCCTTGCTGGTGCTCTTGAAGAGTACAAAGAGAAAGTCGGTGATAACGGTCTTACGGATGATGATATTTACACATTACAGTGATATACTCAAGGCCACTACAGATAGTGGTCTTTATGGATGTCATTGTCTATACG                                                                                                                                                                                                                                                                                                                     <  1:358739/285‑1`  
 `tgtgtataagagacagGTTGGAAGCTGCGCTGGATCAGCGGATGCTTGCGGACCTTTCGGACCATGAGCGTCGCTCTCAGCAACGCTATAATGCTATTAACAAACTGTTAGACCGCCACAAGTTCCAGATTGGTAAGTTGCAGCCGGATGTTCACATCTTAGGTGGGCTTGCTGGTGCTCTTGAAGAGTACAAAGAGAAAGTCGGTTATAACGGTCTTACGGATGATGATATTTACACATTACAGTGATATACTCAAGGCCACTACAGATAGTGGTCTTTATGGATGTCATTGTCTATACG                                                                                                                                                                                                                                                                                                                     <  1:64928/285‑1`  
 `gtgtagcagagaacgcGTTGGACAAGGCGATGGCTCAGCTTATGCTTGCGGACCTTTCGCACCATGAGCGTCGCTCTCCGCAACTCTATAATGCTATTAACAAACTTTTAGACCGCCACAAGTTCCAGATTGGTAAGTTGCAGCCGGATGTTCACATCTTAGGTGGCCTTGCTGGTGCTCTTGAAGAGTACAAAGAGAAAGTCGGTGATAACGGTCTTACGGATGATGATATTTACACATTACAGTGATATACTCAAGGCCACTACAGATAGTGGTCTTTATGGATGTCATTGTCTATACG                                                                                                                                                                                                                                                                                                                     <  1:313506/285‑1`  
 `gtctcggagatgtgtataagagacagGCGATGTCTCAGCGTATGCTTGCGGACCTTTCGGACCATGAGCGTCGCTCTCCGCAACTCTATAATGCTATTAACAAACTGTTAGACCGCCACAAGTTCCAGATTGGTAAGTTGCAGCCGGATGTTCACATCTTAGGTGGCCTTGCTGGTGCTCTTGAAGAGTACAAAGAGAAAGTCGGTGATAACGGTCTTACGGATGATGATATTTACACATTACAGTGATATACTCAAGGCCACTACAGATAGTGGTCTTTATGGATGTCATTGTCTATACG                                                                                                                                                                                                                                                                                                                     <  1:465467/275‑1`  
 `ggtataagagacAGATGTTGGACACTGCGATGGCTCAGCGTATGCTTGCGGACCTTTCGGACCATGAGCGTCGCTCTCCGCAACTCTATAATGCTATTAACAAACTGTTAGACCGCCACAAGTTCCAGATTGGTAAGTTGCAGCCGGATGTTCACATCTTAGGTGGCCTTGCTGGTGCTCTTGAAGAGTACAAAGAGAAAGTCGGTGATAACGGTCTTACGGATGACGATATTTACACATTACAGTGATATACTCAAGGCCACTACAGATAGTGGTCTTTATGGATGTCATTGTCTATACG                                                                                                                                                                                                                                                                                                                     <  1:164663/289‑1`  
 `gagacagCTTAGAGATGTTGGACACTGCGATGGCTCAGCGTATGCTTGCGGACCTTTCGGACCATGAGCGTCGCTCTCCGCAACTCTATAATGCTATTAACAAACTGTTAGACCGCCACAAGTTCCAGATTGGTAAGTTGCAGCCGGATGTTCACATCTTAGGTGGCCTTGCTGGTGCTCTTGAAGAGTACAAAGAGAAAGTCGGTGATAACGGTCTTACGGATGATGATATTTACACATTACAGTGATATACTCAAGGCCACTACAGATAGTGGTCTTTATGGATGTCATTGTCTATACG                                                                                                                                                                                                                                                                                                                     <  1:84963/294‑1`  
 `ctcggagatgtgtataagagacagCTGCGATGGCTCAGCGTATGCTTGCGGACCTTTCAGACCATGAGCGTCGCGCTCCGCAACTCTATAATGCTATTAACAAACTGTTAGACCGCCACAAGTTCCAGATTGGTAAGTTGCAGCCGGATGTTCACATCTTAGGTGGCCTTGCTGGTGCTCTTGAAGAGTACAAAGAGAAAGTCGGTGATAACGGTCTTACGGATGATGATATTTACACATTACAGTGATATACTCAAGGCCACTACAGATAGTGGTCTTTATGGATGTCATTGTCTATACG                                                                                                                                                                                                                                                                                                                     <  1:476078/277‑1`  
 `ctcggagatgtgtataagagaaagCTGCGATGGCTCAGCGTATGCTTGCGGACCTTTCGGACCATGAGCGTCGGTCTCCGCAACTATATAATGCTATTAACAAACTGTTAGACCGCCACAAGTTCCAGAGTGGTAAGTTGCAGCCGGATGTTCACATCTTATGTGGCCTTGCTGGTGCTCTTGAAGAGTACAAAGAGAAAGTCGGTGATAACGGTCTTACGGATGATGATATTTACACATTACAGTGATATACTCAAGGCCACTACAGATAGTGGTCTTTATGGATGTCATTGTCTATACG                                                                                                                                                                                                                                                                                                                     <  1:256292/277‑1`  
 `TTACCTTCTTAGAGATGTTGGACACTGCCGTGGATCAGCGTCTGCTTGAGGAACTTTCGCACCATGAGTGTCGCTATACGCAAGTCTATAATGCTAATAACAAACTGTTAGACCGACATAAGTTCCAGATTGGTAAGTTGCAGCCGGATGTTCACATCTTAGGTTGCCTTGCTGTTGCTCTTGAAGAATACAAAGAGAAAGGCGGTGATAACGGTCTTACGGATGATGATATTTACACATTACAGTGATATACTCAAGGCCACTACAGATAGTGGTCTTTATGGATGTCATTGTCTATACG                                                                                                                                                                                                                                                                                                                     <  1:40874/301‑1`  
 `TTACATTCTTAGGGATGTTGTACACTGGGATGGCTCAGAGTATGCTTGCGGACCTTTCGGACCATGAGCGTCGCTCTCCGCAACTCTATAATGCTATTAACAAACTGTTAGACCGCCATAAGTTCCAGATTGGTAAGTTGCAGCCGGATGTTCACATCTTAGGTGGCCTTGCTGGTGCTATTGAAGAGTACAAAGAGAAAGTCGGTGATAACGGTCTTACGGATGATGATATTTACACATTACAGTGATATACTCAAGGCCACTACAGATAGTGGTCTTTATGGATGTCATTGTCTATACG                                                                                                                                                                                                                                                                                                                     <  1:342669/301‑1`  
 `TTACATTCTTAGGGATGTTGGACACTGCGATGGCTCAGGGTATGCTTGCGGACCTTTCGGACCATGAGCGTCGCTCTCCGCAACTCTATAATGCTATTAACAAACTGTTAGACCGCCACAAGTTCCAGATTGGTAAGTAGCAGCCGGATGTTCACATCTTAGGTGGCCTTGCTGGTGCTCTTGAAGAGTACAAAGAGAAAGTCGGTGATAACGGTCTTACGGATGATGATATTTACACATTACAGTGATATACTCAAGGCCACTACAGATAGTGGTCTTTATGGATGTCATTGTCTATACG                                                                                                                                                                                                                                                                                                                     <  1:378476/301‑1`  
 `TTACATTCTTAGAGATTTTGCACACTGCGATGGCTCAGCGTATGCTTGCGGACCGTGCGTACCATGAGCGTCGCTCTCCGCAACTCTATAATGCTATTAACAAACTGTTAGACCGCCACAAGTTCCAGATTGGTAAGTTGCAGCCGGATGTTCACATCTTAGGTGGCCTTGCTGGTGCTCTTGAAGAGTACAAAGAGAAAGTCGGTGATAACGGTCTTACGGATGATGATATTTACACATTACAGTGATATACTCAAGGCCACTACAGATAGTGGTCTTTATGGATGTCATTGTATATACG                                                                                                                                                                                                                                                                                                                     <  1:292636/301‑1`  
 `TTACATTCTTAGAGATGTTTGACACTGCGATGGCTCAGCGTATGCTTGCGGACCTTTCGGACCATGAGCGTCGCTCTCCGAAACTCTATAATGCTATTAACAAACTGTTAGACCGCCACAAGTTCCAGATTGGTAAGTTGCAGCCGGATGTTCACATCTTAGGTGGCCTTGCTGGTGCTCTTGAAGAGTACAAAGAGACAGTCGGTGATAACGGTCTTACGGATGATGATATTTACACATTACAGTGATATACTCAAGGCCACTACAGATAGTGGTCTTTATGGATGTCATTGTCTATACG                                                                                                                                                                                                                                                                                                                     <  1:117689/301‑1`  
 `TTACATTCTTAGAGATGTTGGACACTGCTATGGCTCAGCGGATGCTTGCGGTCCTTTCGGAACATGCGCGTCGCTCTTCGCACCTCTATAATGCTATTAACAAACTGTTAGACCGCCACAAGTTCCAGATTGGTAAGTTGCAGTCGGATGTTCACATCTTAGGTGGCCTTGCTGGTGCTCTTGAAGAGTACAAAGAGAAAGTCGGTGATAACGGTCTTACGGATGATGATATTTACACATTACAGTGATATACTCAAGGCCACTACAGATAGTGGTCTTTATGGATGTCATTGTCTATACG                                                                                                                                                                                                                                                                                                                     <  1:305756/301‑1`  
 `TTACATTCTTAGAGATGTTGGACACTGCGATGGCTCAGCGTATGCTTGCGGACCTTTCGGACCATGAGCGTCGCTCTCCGCAACTCTATAATGCTATTAACAAACTGTTAGACCGCCACAAGTTCCAGATTGGTAAGTTGCAGCCGGATGTTCACATCTTAGGTGGCCTTGCTGGTGCTCTTGAAGAGTACAAAGAGAAAGTCGGTGATAACGGTCTTACGGATGATGATATTTACACATTACAGTGATATACTCAAGGCCACTACAGATAGTGGTCTTTATGGATGTCATTGTCTATACG                                                                                                                                                                                                                                                                                                                     <  1:430507/301‑1`  
 `TTACATTCTTAGAGATGTTGGACACTGCGATGGCTCAGCGTATGCTTGCGGACCTTTCGGACCATGAGCGTCGCTCTCCGCAACTCTATAATGCTATTAACAAACTGTTAAACCGCCACAAGTTCCAGATTGGTAAGTTGCAGCCGGATGTTCACATCTTAGGTGGCCTTGCTGGTGCTCTTGAAGAGTACAAAGAGAAAGTCGGTGATAACGGTCTTACGGATGATGATATTTACACATTACAGTGATATACTCAAGGCCACTACAGATAGTGGTCTTTATGGATGTCATTGTCTATACG                                                                                                                                                                                                                                                                                                                     <  1:476728/301‑1`  
 `TTACATTCTTAGAGATGTTGGACACTGCGATGGCTCAGCATATGCTTGCGGACCTTGCGGACCATGAGCGTCGCTCTCCGCAACTCTATAATGCTATTAACAAACTGTTAGACCGCCACAAGTTCCAGATTGGTAAGTTGCAGCCGGATGTTAACATCTTAGGTGGCCTTGCTGGTGCTCTTGAAGAGTACAAAGAGAAAGTCGGTGATAACGGTCTTACGGATGATGATATTTACACATTACAGTGATATACTCAAGGCCACTACAGATAGTGGTCTTTATGGATGTCATTGTCTATACG                                                                                                                                                                                                                                                                                                                     <  1:446336/301‑1`  
 `TTACATTCTTAGAGATGTTGGACACTGCGATGACTCAGCGTATGCTTGCGGACCTTTCGGACCATGAGCGTCGCTCTCCGCAACTCTATAATGCTATTAACAAACTGTTAGACCGCCACAAGTTCCAGATTGGTAAGTTGCAGCCGGATGTTCACATCTTAGGTGGCCTTGCTGGTGCTCTTGAAGAGTACAAAGAGAAAGTCGGTGATAACGGTCTTACGGATGATGATATTTACACATTACAGTGATATACTCAAGGCCACTACAGATAGTGGTCTTTATGGATGTCATTGTCTATACG                                                                                                                                                                                                                                                                                                                     <  1:297611/301‑1`  
 `TTACATTCTTAGAGATGTTGGAAACTGCGATGGCTCAGAGTATGCTTGCGGACCTTTCGGACCATGAGCGTCGCTCTCCGCAACTCTATAATGCTATTAACAAACTGTTAGACCGCCACAAGTTCCAGATTGGTAAGTTGCAGCCGGATGTTCACATCTTAGGTGGCCTTGCTGGTGCTCTTGAAGAGTACAAAGAGAAAGTCGGTGATAACGGTCTTACGGATGATGATATTTACACATTACAGTTATATACTCAAGGCCACTACAGATAGTGGTCTTTATTGATGTCATTGTCTATACG                                                                                                                                                                                                                                                                                                                     <  1:347918/301‑1`  
 `TTACATTCTTAGAGATGGTTGACACTTCGATGGCTCAGCGTATGCTTGCGGACCTTTCGGACCATGAGCGTCGCTCTCCGCAACTCTATAATGCTATTAACAAACTGTTAGACCGCCACAAGTTCCAGATTGGTAAGTTGCAGCCGGATGTTCACATCTTAGGTGGCCTTGCTGGTGCTCTTGAAGAGTACAAAGAGAAAGTCGGTGATAACGGTCTTACGGATGATGATATTTACACATTACAGTGATATACTCAAGGCCACTACAGATAGTGGTCTTTATGGATGTCATTGTCTATACG                                                                                                                                                                                                                                                                                                                     <  1:452140/301‑1`  
 `TTACAGTCTTAGAGATGTTGGACACTGCGATTGCTCAGCGTATGCTTGCTGACCTTTCTGACCATGAGCGTCGCTCTCCGCAACTCTATAATGCTATTAACAAACTGTTAGACCGCCACAAGTTCCAGATTGGTAAGTTGCAGCCGGATGTTCACATCTTAGGTGGCCTTGCTGGTGCTCTTGAAGAGTACAAAGAGAAAGTCGGTGATATCGGTCTTACGGATGATGATATTTACACATTACAGTGATATACTAAAGGCCACTACAGATAGTGGTCTTTATGGATGTCATTGTCTATACG                                                                                                                                                                                                                                                                                                                     <  1:205043/301‑1`  
   `gagTTCTTAGAGATGTGGGACTAGTCGATGGCTCAGCGTATGCGTGCGGACCTTTCGAACCATTAGCGGCGCTCTCCGCAACTCTATAATGCTATTAACAAACTGTTAGACCGCCACAAGTTCCAGATTGGGAAGTTGCAGCCGGATGTTCACATCTTAGGTGGCGTTGCGTGTGCTCTTGAAGAGTACAAAGAGAAAGTAGGTGATAACGGTCTTACGGATGATGATATTTACACATTACAGTGATATACTCAAGGCCACTACAGATAGTGGTCTTTATGGATGTCATTGTCTATACGAG                                                                                                                                                                                                                                                                                                                   <  1:484914/298‑1`  
   `gacagCTTAGAGATGTTGGACACTGCGATGGCTCAGCGTATGCTTGCGGACCTTTCGGACCATGAGCGTCGCTCTCCGCAACTCTATAATGCTATTAACAAACTGTTAGACCGCCACAAGTTCCAGATTGGTAAGTTTCAGCCGGATGTTCACATCTTAGGTGGCCTTGCTGGTGCTCTTGAAGAGTACAAAGAGAAAGTCGGTGATAACGGTCTTACGGATGATGATATTTACACATTACAGTGATATACTCAAGGCCACTACCGATAGTGGTCTTTATGGATGTCATTGTCTATACGAG                                                                                                                                                                                                                                                                                                                   <  1:164249/296‑1`  
   `ACATTCTTAGGTATGTTGGAAACTGCGATGGCTCAGCGTATTCTTGCGGACCTTTCGGACCATGAGCGTCGCTCTCCGCAACTCTATAATGCTATTAACAAACTGTTAGACCGCCACAAGTTCCAGATTGGTAAGTTGCAGCCGGATGTTCACATCTTAGGTGGCCTTGCTGGTGCTCTTGAAGAGTACAAAGAGAAAGTCGGTGATAACGGTCTTACGGATGATGATATTTACACATTACAGTGATATACTCAAGGCCACTACAGATAGTGGTCTTTATGGATGTCATTGTCTATACGAG                                                                                                                                                                                                                                                                                                                   <  1:387137/301‑1`  
   `ACATTCTTAGAGATGTTGGACACTGCGATGGCTCAGCGTATGCTTGCGGACCTTTCGGACCATGAGCGTCGCTCTCCGCAACTCTATAATGCTATTAACAAACTGTTCGACCGCCACAAGTTCCAGATTGGTAAGTTGCAGCCGGATGTTCACATCTTATGTGGCCTTGCTGGTGCTCTTGAATAGTACAAAGAGAAAGTCGGTGATAACGTTCTTACGGATGATGATCTTTACACCTTACAGTGATATACTAAAGGCCACTACAGTTATTAGTCTTTATGTATGTCATTGTCTCTACGcg                                                                                                                                                                                                                                                                                                                   >  1:498776/1‑299`  
   `ACATTCTTAGAGATGTTGGACACTGCGATGGCTCAGCGTATGCTTGCGGACCTTTCGGACCATGAGCGTCGCTCTCCGCAACTCTATAATGCTATTAACAAACTGTTAGACCGCCACAAGTTCCAGATTGGTAAGTTGCAGCCGGATGTTCACATCTTAGGTGGCCTTGCTGGTGCTCTTGAAGAGTACAAAGAGAAAGTCGGTGATAACGGTCTTACGGATGATGATATTTACACCTTACAGTGATATACTCAAGGCCACTACAGCTAGTGGTCTTTATGGATGTCATTGTCTATACGAG                                                                                                                                                                                                                                                                                                                   >  1:26300/1‑301`  
   `ACATTCTTAGAGATGTTGGACACTGCGATGGCTCAGCGTATGCTTGCGGACCTTTCGGACCATGAGCGTCGCTCTCCGCAACTCTATAATGCTATTAACAAACTGTTAGACCGCCACAAGTTCCAGATTGGTAAGTTGCAGCCGGATGTTCACATCTTAGGTGGCCTTGCTGGTGCTCTTGAAGAGTACAAAGAGAAAGTCGGTGATAACGGTCTTACGGATGATGATATTTACACATTACAGTGATATACTCAAGGCCACTACAGATAGTGGTCTTTATGGATGTCATTGTCTATACGAG                                                                                                                                                                                                                                                                                                                   <  1:469424/301‑1`  
   `ACATTCTTAGAGATGTTGGACACTGCGATGACTCAGCGTATGCTTGCGGACCTTTCGGACCATGAGCGTCGCTCTCCGCAACTCTATAATGCTATTAACAAACTGTTAGACCGCCACAAGTTCCAGATTGGTAAGTTGCAGCCGGATGTTCACATCTTAGGTGGCCTTGCTGGTGCTCTTGAAGAGTACAAAGAGAAAGTCGGTGATAACGGTCTTACGGATGATGATATTTACACATTACAGTGATATACTCAAGGCCACTACAGATAGTGGTCTTTATGGATGTAATTGTCTATACGAG                                                                                                                                                                                                                                                                                                                   <  1:181887/301‑1`  
   `ACATTCTTAGAGATGGTGGACACTGCGATGGCTCAGCGTAGGCTTGCGGACCTTTCGGACCATTAGCGTCGATATCCGCAACACTATGACGCTATCAACAAACTGTTAGACCGCCACAAGTTTCAGATTGGTAAGTTGCAGACGGATGTTCACATCTTAGGGGGCCTTGCTGGTGCTCTTGAAGAGGACAAATAGAAAGTCGGTGATAACGGTCTTACTGATGATGATATTTACACATTACAGTGATATACTCAAGGCCACTACAGATAGTGGTCTTTATGGATGTCATTGTCTATACGAG                                                                                                                                                                                                                                                                                                                   <  1:110535/301‑1`  
   `ACATGCTTAGAGATGTTGGACACTGCGATGGCTCAGCGTATGCTTGCGGACCTTTCGGACCATGAGCGTCGCTCTCCGCAACTCTATAATGCTATTAACAAACTGTTAGACCGCCACAAGTTCCAGATGGGTAAGTTGCATCCGGATGTTCACATCTTAGGTGGCCTTGCTGGTGCTCTTGAAGAGTACAAAGAGAAAGTCGGTGATAACGGTCTTACGGATGATGATATTTACACATTACAGTGATATACTCAAGGCCACTACAGATAGTGGTCTTTATGGATGTCATTGTCTATACGAG                                                                                                                                                                                                                                                                                                                   <  1:168232/301‑1`  
    `CATTATTAGAGATGTTGGACACTGCGATGGCTCAGCGTATGCTTGCGGACCTTTCGGACCATGAGCGTCGCTCTCCGCAACTCTATAATGCTATTAACAAACTGTTAGACCGCCACAAGTCCAAGATTGGTAAGTTGCAGCCGGATGTTCACATCTTAGGTGGTCTTGCTGGTGCTCTTGAAGAGTACAAAGAGAAAGTCGGTGATAAAGGTCTTACGGATGATGATATTTACACATTACAGTGATATACTCAAGGCCACTACAGATAGTGGTCTTTATGGATGTCATTGTCTATACGAGA                                                                                                                                                                                                                                                                                                                  <  1:240207/301‑1`  
     `ATTCTTAGAGATTTTGGACACTGCGATGGCTCAGCGTATGCTTGCGGACCTTTCGGACCATGAGCGTCGCTCTCCGAAACTCTATAATGCTATTAACAAACTGTTAGACCGCCACAAGTTCCAGATTGGTAAGTTGCAGCCGGATGTTCACATCTTAGGTGGCCTTGCTGGTGCTCTTGAAGAGTACAAAGAGAAAGTCGGTGATAACGGTCTTACGGATGATGATATTTACACATTACAGTGATATACTCAAGGCCACTACAGATAGTGGTCTTTATGGATGTCATTGTCTATACGAGAT                                                                                                                                                                                                                                                                                                                 <  1:132416/301‑1`  
     `ATTCTGAGAGATGTATGACACTGCGATGGCTCAGCGTATGCTTGCTGACCTTTCGGACCATGAGCGTCGCTCTCCGCAACTCTATAATGCTATTAACAAACTGTTAGACCGCCACAAGTTCCAGATTGGTAAGTTGCAGCCGGATGTTCACATCTTAGGTGGCCTTGCTGGTGCTCTTGAAGAGTACAAAGAGAAAGTCGGTGATAACGGTCTTACGGATGATGATATTTACACATTACAGTGATATACTCAAGGCCACTACAGATAGTGGTCTTTATGGATGTCATTGTCTATACGAGAT                                                                                                                                                                                                                                                                                                                 <  1:355286/301‑1`  
      `TTCTTATAGATGTTGGACACTGCGATGGCTCAGCGTATGCTTGCGGACCTTTCGGACCATGAGCGTCGCTCTCCGCAACTCTATAATGCTATTAACAAACTGTTAGACCGCCACAAGTTCCAGATTGGTAAGTTGCAGCCGGATGTTCACATCTTAGGTGGCCTTGCTGGTGCTCTTGAAGAGTACAAAGAGAAAGTCGGTGATAACGGTCTTACGGATGATGATATTTACACATTACAGTGATATACTCAAGGCCACTACAGATAGTGGTCTTTATGGATGTCATTGTCTATACGctgtc                                                                                                                                                                                                                                                                                                                >  1:145162/1‑296`  
      `TTCTTAGAGATGTTGGACACTGCGATGGCTCAGCGTATGCTTGCGGACCTTTCGTACCATGAGCGTCGATCTCCGCAACTCTATAATGCTATTAACAAACTGTTAGACGGCCACAAGTTCCAGATTGGTAAGTTGCAGCCGGATGTTCACATCTTAGGTGGCCTTGATGGTGCTCTTGAAGAGTACAAAGAGAAAGTCGGTGATAACGGTCTTACGGATGATGATATTTACACATTACAGTGATATACTCAAGGCCACTACAGATAGTGGTCTTTATGGATGTAATTGTCTATACGAGATG                                                                                                                                                                                                                                                                                                                >  1:412755/1‑301`  
      `TTCTTAGAGATGTTGGACACTGCGATGGCTCAGCGTATGCTTGCGGACCTTTCGGACCATGAGCGTCGCTCTCCGCAACTCTATAATGCTATTAACAAACTGTTAGACCGCCCCCAGTTCCAGATTGGTAAGTTGCAGCCGGATGTTCACATCTTAGGTGGCCTTGCTGGTGCTCTTGAAGAGTACAAAGAGAAAGTCGGTGATAACGGTCTTACGGATGATGCTATTTACACATTACAGTGATATACTCAAGGCCACTACAGATAGTGGTCTTTATGGATGTCATTGTTTATCAGAGAat                                                                                                                                                                                                                                                                                                                >  1:226339/1‑299`  
      `TTCTTAGAGATGTTGGACACTGCGATGGCTCAGCGTATGCTTGCGGACCTTTCGGACCATGAGCGTCGCTCTCCGCAACTCTATAATGCTATTAACAAACTGTTAGACCGCCACAAGTTCCAGATTGGTAAGTTGCAGCCGGATGTTCACATCTTAGGTGGCCTTGCTGGTGCTCTTGAAGAGTACAAAGAGAAAGTCGGTGATAACGGTCTTACGGATGATGATATTTACACATTACAGTGATATACTCAAGGCCACTACAGATAGTGGTCTTTATGGATGTCATTGTCTATACGctgtc                                                                                                                                                                                                                                                                                                                >  1:427954/1‑296`  
      `TTCTTAGAGATGTTGGACACTGCGATGGCTCAGCGTATGCTTGAGGACCTTTCGGACCATGAGCGTCGCTCTCCGCAACTCTATAATGCTATTAACAAACTGTTAGACCGCCACAAGTTCCAGATTGGTAAGTTGCAGCCGGATGTTCACAACATAGGTGGCCTTGCTGGTGCTCTTGAAGAGTACAAAGAGAAAGTCGGTGATAACGGTCTTACGGATGATGATATTTACACATTACAGTGATACACTCAAGTCTACTACAGATAGTGGTCTTTATGGATGCCATTGCCTATACGctgtt                                                                                                                                                                                                                                                                                                                >  1:22124/1‑296`  
      `TTCTTAGAGATGTTGGACACTGCGATGGCTCAGCGTATGATTGCGGACCTTTCGGACCATGAGCGTCGCTCTCCGAAACTCTATAATGCTATTAACAAACTGTTAGACCGCCACAAGTTCCAGATTGGTAAGTTGCAGCCGGATGTTCACATCTTAGGTGGCCTTGCTGGTGCTCTTGAAGAGTACAAAGAGAAAGTCGGTGATAACGGTCTTACGGATGATGATATTTACACATTACAGTGATCTACTCAAGGCCACTACAGATAGTGGTCTTTATGGATGTCATTGTCTATACGAGATG                                                                                                                                                                                                                                                                                                                >  1:246351/1‑301`  
       `TCTTTGGGATGTTGGACACTGCGATGGCTCAGGGTATGCTTGCGGACCTTTCGGACCATGAGCGTCGCTCTCCGCAACTCTATAATGCTATTAACAAACTGTTAGACCGCCACAAGTTCCAGATTGGTAAGTTGCAGCCGGATGTTCACATCTTAGGTGGACTTGCTGGTGCTCTTGAAGAGTACAAAGAGAAAGTCGGTGATAACGGTCTTACGGATGATGATATTTACACATTACAGTGATATACTCAAGGCCACTACAGATAGTGGTCTTTATGGATGTCATTGTCTATACGAGATGC                                                                                                                                                                                                                                                                                                               <  1:421910/301‑1`  
       `TCTTAGATATGTGGGACACTGCGATGGCTCAGAGTATGCTTGCGGACCTTTCGGACCATGAGCGTCGCTCTCCGCAACTCTATAATGCTATTAACAAACTGTTAGACCGCCACAAGTTCCAGATTGGTAAGTTGCAGCCGGATGTTCACATCTTAGGTGGCCTTGCTGGTGCTCTTGAAGAGTACAAAGAGAAAGTCGGTGATAACGGTCTTACGGATGATGATATTTACACATTACAGTGATATACTCAAGGCCACTACAGATAGTGGTCTTTATGGATGTCATTGTCTATACGAGATGC                                                                                                                                                                                                                                                                                                               <  1:61677/301‑1`  
        `CTTATAGATGTTGGACACTGCGATGGCTCAGCGTATGCTTGCGGACCTTTCGGACCATGAGCGTCGCTCTCCGCAACTCTACAATGCTATTAACAAACTGTTAGAACGCCACAAGTCCACGATTGGTAAGTTGCAGTAGGATGTTCACATGTAAGGTGGCCTTGTTGGTGCTCTTGAAGAGTACAAATAGTAAGTCGGTGATAACGGTCTTACGGATGAAGATATTTACACATTTCAGTGATATACTTTATGCCACTACAGAGAGTGGTCCTTATGGATGTATTTGACTATACGTGATGCT                                                                                                                                                                                                                                                                                                              >  1:44496/1‑301`  
        `CTTAGAGATGTTGGACACTGCGATGGCTCAGCGTATGCTTGCGGACCTTTCGGACCATGAGCGTCGCTCTCCGCAACTCTATAATGCTATTAACAAACTGTTAGACCGCCACAAGTTCCAGATTGGTAAGTTGCAGCCGGATGTTCACATCTTAGGTGGCCTTGCTGGTGCTCTTGAAGAGTACAAAGAGAAAGTCGGTGATAACGGTCTTACGGATGATGATATTTACACATTACAGTGATATACTCAAGGCCACTACAGATAGTGGTCTTTATGGATGTCATTGTCTATACGAGATGCT                                                                                                                                                                                                                                                                                                              >  1:214413/1‑301`  
        `CTTAGAGATGTTGGACACTGCGATGGCTCAGCGTATGCTAGCGGACCTTTCGGACCATGAGCGTCGCTCTCCGCAACTCTATAATGCTATTAACAAACTGTTAGACCGCCACAAGTTCCAGATTGGTAAGTTGCAGCCGGATGTTCACATCTTAGGTGGCCTTGCTGGTGCTCTTGAAGAGTACAAAGAGAAAGTCGGTGATAACGGTCTTACGGATGATGATATTTACACATTACAGTGATATACTCAAGGCAACTACAGATAGTGGTCTTTATGGATGTCATTGTCTATACGAGCTGtc                                                                                                                                                                                                                                                                                                              >  1:433112/1‑299`  
         `TTAGAGATGTTGGACACTGCGATGGCTCAGCGTATGCTTGCGGACCTTTCGGACCATGAGCGTCGATCTCCGCAACTCTATAATGCTATTAACAAACTGTTCGACCGCCACAAGTTCCAGATTGGTAAGTTGCAGCCGGATGTTCACATCTTAGGTGGCCTTGCTGGTGCTCTTGAAGAGTACAAAGAGAAAGTCGGTGATAACGGTCTTACGGATGATGATATTTACACATTACAGTGATATACTCAAGGCCACTACAGATAGTGGTCTTTATGGATGTCATTGTCTATACGAGCTGtct                                                                                                                                                                                                                                                                                                             >  1:162328/1‑298`  
          `TAGAGATGTTGGACACTGCGATGGCTCAGCGTATGCTTGCGGACCTTTCGGACCATGAGCGTCGCTCTCCGCAACTCTATAATGCTATTAACAAACTGTTAGACCGCCACAAGTTCCAGATTGGTAAGTTGCAGCCGGATGTTCACATCTTAGGTGGCCTTGCTGGTGCTCTTGAAGAGTACAAAGAGAAAGTCGGTGATAACGGTCTTACGGATGATGATATTTACACATTACAGTGATATACTCAAGGCCACTACAGATAGTGGTCTTTATGGATGTCATTGTCTATACGAGATctgtc                                                                                                                                                                                                                                                                                                            >  1:185882/1‑296`  
            `GAGATGTTGGACACTGCGATGGCTCAGCGTATGCTTGCGGACCTTTCGGACCATGAGCGTCGCTCTCCGCAACTCTATAATGCTATTAACAAACTGTTAGACCGCCACAAGTTCCAGATTGGTAAGTTGCAGCCGGATGTTCACATCTTAGGTGGCCTTGCTGGTGCTCTTGAAGAGTACAAAGAGAACGCCGGTGATAACGGTCTTACGGATGATGATATTTACACATTACAGTGATATACTCAAGGCCACTACATATATTGGTCTTTATGGATGTCATTGTCTATACGctgtctcttat                                                                                                                                                                                                                                                                                                          >  1:301726/1‑290`  
               `ATGTTGGACACTGCGATGGCTCAGCGTATGCTTGCGGACCTTTCGGACCATGAGCGTCGCTCTCCGCAACTCTATAATGCTATTAACAAACTGTTAGACCGCCACAAGTTCCAGATTGGTAAGTTGCAGCCGGATGTTCACATCTTAGGTGGCCTTGCTGGTGCTCTTGAAGAGTACAAAGAGAAAGTCGGTGATAACGGTCTTACGGATGATGATATTTACACATTACAGTGATATACTCAAGGCCACTACAGATAGTGGTCTTTATGGATGTCATTGTCTATACGctgtctcttataca                                                                                                                                                                                                                                                                                                       >  1:291809/1‑287`  
                 `GTTGGACACTGCGATGGCTCAGCGTATGCTTGCGGACCTTTCGGACCATGAGCGTCGCTCTCCGCAACTCTATAATGTTATTAACAAACTGTTAGACCGCCACAAGTTCCAGATTGGTAAGTTGCAGCCGGATGTTCACATCTTAGGTGGCCTTGCTGGTGCTCTTGAAGAGTACAAAGAGAAAGTCGGTGATAACGGTCTTACGGATGATGATATTTACACATTACAGTGATATACTCAAGGCCACTACAGATAGTGGTCTTTATGGATGTCATTGTCTATACGctgtctcttatacaca                                                                                                                                                                                                                                                                                                     >  1:424402/1‑285`  
                 `GTTGGACACTGCGATGGCTCAGCGTATGCTTGCGGACCTTTCGGACCATGAGCGTCGCTCTCCGCAACTCTATAATGCTATTAACAAACTGTTAGACCGCCACAAGTTCCAGATTGGTAAGTTGCAGCCGGATGTTCACATCTTAGGTGGCCTTGCTGGTGCTCTTGAAGAGTACAAAGAGAAAGTCGGTGATAACGGTCTTACGGATTATGATATTTACACATTACAGTGATATACTCAAGGCCACTACAGATAGTGGTCTTTATGGATGTCATTGTCTATACGctgtctcttatacaca                                                                                                                                                                                                                                                                                                     >  1:250896/1‑285`  
                 `GTTGGACACTGCGATGGCTCAGCGTATGCTTGCGGACCTTTCGGACCATGAGCGTCGCTCTCAGCAACTCTATAATGCTATTAACAAACTGTTAGACTGCCACAAGTTCCAGCTTGGTAAGTTGCAGCCGGGTGTTCACATCTTAGGTGAGCTTTCTGGTGCTTTTGAAGAGTACAAGGAGAAAGTAGGTGATAACGGTCTTACGGTTGATTATTTTTACACATTAGAGTGATATACTCAAGGCCACTACAGATAGTGGTCTTTATGGATGTCATTTTCTAAACGAGtggctccgacggta                                                                                                                                                                                                                                                                                                     >  1:489460/1‑287`  
                         `CTGCGATGGCTCAGCGTATGCTTGCGGACCTTTCGGACCATGAGCGTCGCTCTCCGCAACTCTATAATGCTATTAACAAACTGTTAGACCGCCACAAGTTCCAGATTGGTAAGTTGCAGCCGGATGTTCACATCTTAGGTGGCCTTGCTGGTGCTCTTGAAGAGTACAAAGAGAAAGTCGGTGATAACGGTCTTACGGATGATGATATTTACACATTACAGTGATATACTCAAGGCCACTACAGATAGTGGTCTTTATGGATGTCATTGTCTATACGAGATctgtcgcttatacacatctc                                                                                                                                                                                                                                                                                             >  1:307865/1‑281`  
                           `GCGATGGCTCAGCGTATGCTTGCGGACCTTTCGGACCATGAGCGTCGCTCTCCGCAACTCTATAATGCTATTAACAAACTGTTAGACCGCCACAAGTTCCAGATTGGTAAGTTGCAGCCGGATGTTCACATCTTAGGTGGCCTTGCTGGTGCTCTTGAAGAGTACAAAGAGAAAGTCGGTGATAACGGTCTTACGGATGATGATATTTACACATTACAGTGATATACTCAAGGCCACTACAGATAGTGGTCTTTATGGATGTCATTGTCTATACGAGCTGtctcttatacccatctccgag                                                                                                                                                                                                                                                                                           >  1:438934/1‑280`  
                            `CGATGGCTCAGCGTATGCTTGCGGACCTTTCGGACCATGAGCGTCGCTCTCCGCAACTCTATAATGCTATTAACAAACTGTTAGACCGCCACAAGTTCCAGATTGGTAAGTTGCAGCCGGATGTTCACATCTTAGGTGGCCTTGCTGGTGCTCTTGAAGAGTACAAAGAGAAAGTCGGTGATAACGGTCTTACGGATGATGATATTTACACATTACAGTGATATACTCAAGGCCACTACAGATAGTGGTCTTTATGGATGTCATTGTCTATACGctgtctcttatacacatctccgagccc                                                                                                                                                                                                                                                                                          >  1:462164/1‑274`  
                                                     `CCTTTCGGACCATGAGCGTCGCTCTCCGCAACTCTATAATGCTATTAACAAACTGTTAGACCGCCACAAGTTCCAGATTGGTAAGTTGCAGCCGGATGTTCACATCTTAGGTGGCCTTGCTGGTGCTCTTGAAGAGTACAAAGAGAAAGTCGGTGATAACGGTCTTACGGATGATGATATTTACACATTACAGTGATATACTCAAGGCCACTACAGATAGTGGTCTTTATGGATGTCATTGTCTATACGAGATGCTAAACGTATTCTCGCAGTGACCCAGAAGGGTGACGCATGGATTCGT                                                                                                                                                                                                                                                                 >  1:147232/1‑301`  
                                                                              `agagacaTCTATAATGCTATTAACAAACTGTGAGACCGCCACAAGTTCCAGATTGGTAAGTTGCAGCCGGATGTTCACATCTTAGGTGGCCTTGCTGGTGCTCTTGAAGAGTACAAAGAGAAAGTCGGTGATAACGGTCTTACGGATGATGATATTTACACATTACAGTGATATACTCAAGGCCACTACAGATAGTAGTCTTTATGGATGTCATTGTCTATACGAGATGCTAAACGTATTCTCGCAGTGACCAAGAAGGGTGACGCATGGATTCGTGCGTTACAGGATACTATTCGTGAAC                                                                                                                                                                                                                                        <  1:102611/294‑1`  
                                                                                   `ACTCTATAATGCTATTAACAAACTGTTAGACCGCCACAAGTTACAGATTGGTAAGTTGCAGCCGGATGTTCACATCTTAGGTGGCCTTGCTGGTGCTCTTGAAGAGTACAAAGAGAAAGTCGGTGATAACGGTCTTACGGATGATGCTATTTACACATTACAGTGATATACTCACGGCCACTACAGATAGTGGTCTTTATGGATGTCATTGTCTATACGAGATGCTAAACGTATTCTCGCAGTGACCCAGAAGGGTGACGCATGGATTCGTGCGTTACAGGATACTATTCGTTACCTGCAA                                                                                                                                                                                                                                   >  1:265182/1‑301`  
                                                                                                                                     `GTAAGTTGCAGCCGGATGTTCACATCTTAGGTGGCCTTGCTGGTGCTCTTGAAGAGTACAAAGAGAAAGTCGGTGATAACGGTCTTACGGATGATGATATTTACACATTACAGTGATATACTCAAGGCCACTACAGATAGTGGTCTTTATGGATGTCATTGACTATACGAGATGCTAAACGTATTCTCGCAGTGACCCAGAAGGGTGACGCATGGATTCGTGCGTTACAGGATACTATTCGTGAACTGCAACGTAAGTAGGAAATCAAGTAAGGAGGCAATGTGTCTACTCAATCCAATCG                                                                                                                                                                                 <  1:362535/301‑1`  
                                                                                                                                                                                                             `GTGCTAACGGTCTTACGGATGATGATATTTACACATTACAGTGATATACTCAAGGCCACTACAGATAGTGGTCTTTATGGATGTCATTGTCTATACGAGATGCTAAACGTATTCTCGCAGTGACCCAGAAGGGTGACGCATGGATTCGTGCGTTACAGGATACTATTCGTGAACTGCAACGTAAGTAGGAAATCAAGTAAGGAGGCAATGTGTCTACTCAATCCAATCGTAATGCGCTCGTAGTGGCGCAACTGAAAGGAGACTTCGTGGCGTTCCTATTCGTCTTATGGAAGGCGCTAAA                                                                                                         >  1:173972/1‑301`  
                                                                                                                                                                                                                                        `TTTACACATTAAAGGGATATACTCAAGGCCACTACAGATAGTGGTCTTTATGGATGTCATTGTCTATACGGGATGCTAAACGTATTCTCGCAGTGACCCAGAAGGGTGACGCATGGATTCGTGCGTTACAGGATACTATTCGTGAACTGCAAAGTAAGTAGGAAATCAAGTAAGGAGGCAATGTGTCTACTCAATCCAATCGTAATGCGCTCGTAGTGGCGCAACTGAAAGGAGACTTCGTGGCGTTCCTATTCGTCTTATGGAAGGCGCTAAACCTACCGGTGCCCACTAAGTGTCAGAT                                                                              <  1:320438/301‑1`  
                                                                                                                                                                                                                                                                                              `agtgtataagagacaGAGATGCTAAACGTATTCGCGCAGTGACCCAGAAAGGTGACGCATGGATTCGTGCGTTACAGGATACTATTCGTGAACTGCAACGTAAGTAGGAAATCAAGTAAGGAGGCAATGTTTCTACGCAATCCAATCGTAATGCGCTCGTAGTGGCGCAACTGAAAGGAGACTTTGTGGCGTTCCTATTCGTCTTATGGAAGGCGCTAAACCTACCGGTGCCCACTAAGGGTCAGATTGACATGGCTAAGGAGCTGGCGAATGGAGACAACAAGAAGTTCATCTTACAGGC                        <  1:1993/286‑1`  
                                                                                                                                                                                                                                                                                                     `GTCTATACGAGATGCTAAACGTATTCTCGCAGTGACCCAGAAGGGTGACGCATGGATTCGTGCGTTACAGGATACTATTCGTGAACTGCAACGTAAGTAGGAAATCAAGTAAGGAGGCAATGTGTCTACTCAATCCAATCGTAATGCGCTCGTAGTGGCGCAACTGAAAGGAGACTTCGTGGCGTTCCTATTCGTCTTATGGAAGGCGCTAAACCTACCGGTGCCCACTAAGTGTCAGATTGACATGGCTAAGGTGCTGGCGAATGGAGACAACAAGAAGTTCATCTTACAGGCTTTCCGc                 >  1:121694/1‑300`  
                                                                                                                                                                                                                                                                                                      `gccgaACGAGATGCTAAACGTATTCTCGCAGTGACCCAGAAGGGTGACGCATGTATTCGTTCGTTACAGGATACTATTCGTGACCTGCAACGTAAGTAGGAAATCAAGTAAGGAGGCAATGTGTCTACTCAATCCAATCGGAATGCGCTCTTAGTGGCGCAACTGAAAGTAGACTTCGTGGCGTTCCTATTCGTCTTATGGAAGGCGCTAAACCTACCGTTGCCCACTAAGTGTCAGATTGACATGGCTAAGGTGCTGGCGAATGGAGACAACAAGAAGTTCATCTTACAGGCTTTCCGTG                <  1:88740/296‑1`  
                                                                                                                                                                                                                                                                                                       `ataagagacaggaTAAACGTATTCTCGCAGTGACCCATAAGGGTGACGCATGGATTCGTGAGTTACAGGATACTATTCGTGAACTGCAACGTAAGTAGGAAATAAAGTAAGGAGGCAATGTGTCTACTCAATCCAATCGTAATGCGCTCGTAGTGGCGCAACTGAAAGGAGACTTCGTGGCGTTCCTATTCGTCTTATGGAAGGCGCTAAACCTACCGGTGCCCACTAAGTGTCAGATTTACATGGCTAAGGTGCTGGCGAATGTAGACAACAAGAAGTTCATCTTACAGGCTTTCCGTGG               <  1:426242/288‑1`  
                                                                                                                                                                                                                                                                                                        `taagagacagGCTAAACGTATTCTCGCAGTGACCCAGAAGGATGACGAATGGATACGTGCGTTACAGGATACTATTCGTGAACTGCAACGTAAGTAGGAAATCAAGTAAGGAGGCAATGTGTCTACTCAATCCAATCGTAATGCACTCGTAGTGGCGCAACTGAAAGGAGACTTCGTGGCGTTCCTATTCGTCTTATGGAAAGCGCTAAACCTACCGGTGCCCACTAAGTGTCAGATTGACATGGCTAAGGTGCTGGCGAATGGAGACAACAAGAAGTTCATCTTTCAGGCTATCCGTGGT              <  1:392198/291‑1`  
                                                                                                                                                                                                                                                                                                        `ctaccgagaatgTAAATGTATTCTCGAAGGGACCCTGATGGGTGACGAATGGATTCGTGCCTTACAGGAGACTAGTCGTGAACTGAAACGTAAGTAGGAAATCAAGTAAGGAGGTAATGTGTCTACTCAATCCAATCGTAATGCGCTCGTAGTGGCGCAACTGAAAGGAGACTTCGTGGCGTGCCTATTCGTCTTATGGAAGGCGCTAAACAAAACGGTGCCCACTAAGTGTCAGATTGACATGGCTAAGGTGCTGGCGAATGGAGACAACAAGAAGTTCATCTTACAGGCTGTCCGTGGT              <  1:208163/289‑1`  
                                                                                                                                                                                                                                                                                                         `gacctgaattCTAAAAGTAATACCGCAGTGAACCAGAAGGGTGACCCATGGATTCGTGCGTTACAGGATACTATTCGTGAACTGCAACGTAAGTAGGAAATCAAGTAAGTAGGCAATGTGTCTACTCAATCCAATCGTAATGCGCTCGTAGTGGCGCAACTGAAAGGAGACTTCGTGGCGTTCCTATTCGTCTTATGGAAGGCGCTAAACCTACCGGTGCCCACTAAGTGTCAGATTGACATGGCTAAGGTGCTGGCGAATGGAGACAACAAGAAGTTCATCTTACAGGCTTTCCGTGGTA             <  1:15285/291‑1`  
                                                                                                                                                                                                                                                                                                               `aATGCTAAACGTATTCTCGCAGTGACCCATAAGGGTGACGCATGGATTCGTGCGTTACAGGATACTATTCGTGAACTGCAACGTAAGTAGGAAATCAAGTAAGGAGGCAATGTGTCTACTCAATCCAATCGTAATGCGCTCGTAGTGGCGCAACTGAAAGGAGACTTCGTGGCGTTCCTATTCGTCTTATGGAAGGCGCTAAACCTACCGGTGCCCACTAAGTGTCAGATTGACATGGCTAAGGTGCTGGCGAATGGAGACAACAAGAAGTTCATCTTACAGGCTTTCCGTGGTATCGGTA       <  1:317354/300‑1`  
                                                                                                                                                                                                                                                                                                               `aATGCTAAACGGATTCTACAAATGACCCAGAAGGGGGAAGCCTGGATTAGTGCTTTACAGGATACTATTCGTGAACTGCAACGTAAGTAGGAAATCAAGTAAGGAGGCAATTTGTCTACTCAACCCAATCGTAATGCGCTCGTATTGGCGCAACTGAAAGGAGACTTCGTGGCGTTCCTATTCGTCTTATGGAAGGCGCTAAACCTACCGGTGCCCACTAAGTGTCAGATTGACATGGCTAAGGTGCTGGCGAATGGAGACAACAAGAAGTTCATCTTACAGGCTTTCCGTGGTATCAGTA       <  1:509952/300‑1`  
                                                                                                                                                                                                                                                                                                               `GATGCTAAAGGTATACTCGCAGTGACCAAGAAGGGTGACGCATGGATTCGTGCGTTACAGGATAGTATTAGGGAAATGCAACGTAAGTAGGAAATCAAGTAAGGAGGCAATGTGTCTACTCAATCCAATCGTAATGCGCGCGTAGTGGCGCAACTGAAAGGAGACTTCGTGGCGTTCCTATTCGTCTTATGGAAGGCGCTAAACCTACCGGTGCCCACTAAGTGTCAGATTGACATGGCTAAGGTGCTGGCGAATGGAGACAACAAGAAGTTCATCTTACAGGCTTTCCGTGGTATCAGTA       <  1:231617/301‑1`  
                                                                                                                                                                                                                                                                                                               `GATGCTAAACGTATTCTCGCAGTGACCCAGAAGGGTGACGCATGGATTCGTGCGTTACAGGATACTATTCGTGAACTGCAACGTAAGTAGGAAATCAAGTAAGGAGGCAATGTGTCTACTCAATCCAATCGTAATGCTCTCGTAGTGGCGCAACTGAAAGGATACTTCGTGGCGTTCCTATTCGTCTTATGGAAGGCGCTAAACCTACCGGTGCCCACTAAGTGTCAGATTGACATGGCTAAGGTGCTGGCGAATGGAGACAACAAGAAGTTCATCTTACAGGCTTTCCGTGGTATCGGTA       >  1:333246/1‑301`  
                                                                                                                                                                                                                                                                                                               `GATGCTAAACGTATTCTCGCAGTGACCCAGAAGGGTGACGCAGGGATTCGTGCGTTACAGGATACTATTCGTGAACTGCAACGTAAGTAGGAAATCAAGTAAGGAGGCAATGTGTCTACTCAATCCAATCGTAATGCGCTCGTAGTGGCGCAACTGAAAGGAGACTTCGTGGCGTTCCTATTCGTCTTATGGAAGGCGCTAAACCTACCGGTGCCCACTAAGTGTCAGATTGACATGGCTAAGGTGCTGGCGAATGGAGACAACAAGAAGTTCATCTTACAGGCTTTCCGTGGTATCGGTA       <  1:502978/301‑1`  
                                                                                                                                                                                                                                                                                                                 `TGCTAAACGTATTCTCGCAGTGACCCAGAAGGGTGACGCATGGATTCGTGCGTTACAGGATACTATTCGTGAACTGCAACGTAAGTAGGAAATCAAGTAAGGAGGCAATGTGTCTACTCAATCCAATCGTAATGCGCTCGTAGTGGCTCAACTTAAAGGAGACTTCGTGGCGTTCCTATTCGTCTTATGGAAGGCGCTAAACCTACCGGTGCCCACTAAGTGTCAGATTGACATGGCTAAGGTGCTGGCGAATGGAGACAACAAGAAGTTCATCTTACAGGCTTTCCGTGGTATCGGTAAG     >  1:456436/1‑301`  
                                                                                                                                                                                                                                                                                                                 `TGCTAAACGTATTCTCGCAGTGACCCAGAAGGGTGACGCATGGATTCGTGCGTTACAGGATACTATTCGTGAACTGCAACGTAAGTAGGAAATCAAGTAAGGAGGCAATGTGTCTACTCAATCCAATCGTAATGCGCTCGTAGTGGCGCAACTGAAAGGAGACTTCGTGGCGTTCCTATTCGTCTTATGGAAGACGCTAAACCTACCGGTGCCCACTAAGTGTCAGATTTACATGGCTAAGGTGCTGGCGAATGGAGACAACAAGAAGTTCATCTTACATGCTTTCCGTGGTATCGGTAAG     >  1:371103/1‑301`  
                                                                                                                                                                                                                                                                                                                 `TGCTAAACGTATTCTCGCAGTGACCCAGAAGGGTGACGCATGGATTCGTGCGTTACAGGATACTATTCGTGAACTGCAACGTAAGTAGGAAATCAAGTAAGGAGGCAATGTGTCTACTCAATCCAATCGTAATGCGCTAGTAGTGGCGCAACTGAAAGGAGACTTCGTGGCGTTCCTATTCGTCTTATGGAAGGCGCTAAACATACCGGTGCCCACTAAGTGTCAGATTGACATGGCTAAGGTGCTGGCGAATGGAGACAACAAGAAGTTCATCTTACAGGCTTTCCGTGGTATCGGTAAG     >  1:280433/1‑301`  
                                                                                                                                                                                                                                                                                                                 `TGCTAAACGTATTCTCGCAGTGACCCAGAAGGGTGACGCATGGATTCGTGCGATACAGTATACTATTCGTGAACTGCAACGTAAGTAGGAAATCAAGTAAGGAGGCAATGTGTCTACTCAATCCAATCGTAATGCGCTCGTAGTGGCGCAACTGAAAGGAGACTTCGTGGCGTTCCTATTCGTCTTATGGAAGGCGCTAAACCTACCGGTGCCCACTAAGTGTCATATTGACATGGCTAAGGTGCTGGCGAATGGAGACAACAAGAAGTTCATCTTACAGGCTTTCCGTGGTATCGGTctg     >  1:57250/1‑298`  
                                                                                                                                                                                                                                                                                                                 `TGCTAAACGTATTCTAGCAGTGACCCAGAAGGGTGACGCATGGATTAGTGCGTTACAGGATACTATTCGTGAACTGCAACGTAAGCAGGAAATCAAGTAAGGAGGCAATGTGTCTACTCAATCCAATCGTAATGCGCTCGTAGTGGCGCAACTGAAAGGAGACTTCGTGGCGTTCCTATTCGTCTTATGGAAGGCGCTAAACCTACCGGTGCCCACTAAGTGTCAGATTGACATGGCTAAGGTGCTGGCGAATGGAGACAACAAGAAGTTCATCTTACAGGCTTTCCGTGGTATCGGTAAG     >  1:189344/1‑301`  
                                                                                                                                                                                                                                                                                                                  `GCTAAACGTATTCTCGCAGTGACCCAGAAGGGTGACGCATGGATTCGTGCGTTACAGGATACTATTCGTGAACTGCAACGTAAGTAGGAAATCAAGTAAGGAGGCAATGTGTCTACTCAATCCAATCGTAATGCGCTCGTAGTGGCGCAACTGAAAGGAGACTTCGTGGCGTTCCTATTCGTCTTATGGAATGCGCTAAACCTACCGGTGCCCACTAAGTGTCAGATTGACATGGCTCAGGTGCTGGCGAATGGAGACAACAAGAAGTTCATCTTACAGGCTTTCCGTGGTATCGGTAAGT    >  1:234820/1‑301`  
                                                                                                                                                                                                                                                                                                                  `GCTAAACGTATTCTCGCAGTGACCCAGAAGGGTGACGCATGGATTCGTGCGTTACAGGATACTATTCGTGAACTGCAACGTAAGTAGGAAATCAAGTAAGGAGGCAATGTGTCTACTCAATCCAATCGTAATGCGCTCGTAGTGGCGCAACTGAAAGGAGACTTCGTGGCGTTCCTATTCGTCTTATGGAAGGCGCTAAACCTACCGGTGCCCACTAAGTGTCAGATTGACATGGCTAAGGTGCTGGCGAATGGAGACAACAAGAAGTTCATCTTACAGGCTTTCCGTGGTATCGGTCAGT    >  1:459273/1‑301`  
                                                                                                                                                                                                                                                                                                                  `GCTAAACGTATTCTCGCAGTGACCCAGAAGGGTGACGCATGGATTCGTGCGTTACAGGATACTATTCGTGAACTGCAACGTAAGTAGGAAATCAAGTAAGGAGGCAATGTGTCTACTCAATCCAATCGTAATGCGCTCGTAGTGGCGCAACTGAAAGGAGACTTCGTGGCGTTCCTATTCGTCTTATGGAAGGCGCTAAACCTACCGGTGCCCACTAAGTGTCAGATTGACATGGCTAAGGTGCTGGCGAATGGAGACAACAAGAAGTTCATCTTACAGGCTTTCCGTGGTATCGGTActg    >  1:328453/1‑298`  
                                                                                                                                                                                                                                                                                                                  `GCTAAACGTATTCTCGCAGTGACCCAGAAGGGTGACGCATGGATTCGTGCGTTACAGGATACTATTCGTGAACTGCAACGTAAGTAGGAAATCAAGTAAGGAGGCAATGTGTCTACTCAATCCAATCGTAATGCGCTCGTAGTGGCGCAACTGAAAGGAGACTTCGTGGCGTTCCTATTCGTCTTATGGAAGGCGCTAAACCTACCGGTGCCCACTAAGTGTCAGATTGACATGGCTAAGGTGCTGGCGAATGGAGACAACAAGAAGTTCATCTTACAGGCTTTCCGTGGTATCGGTActg    >  1:136238/1‑298`  
                                                                                                                                                                                                                                                                                                                  `GCTAAACGTATTCTCGCAGTGACCCAGAAGGGTGACGCATGGATTCGTGCGTTACAGGATACTATTCGTGAACTGCAACGTAAGTAGGAAATCAAGTAAGGAGGCAATGTGTCTACTCAATCCAATCGTAATGCGCTCGTAGTGGCGCAACTGAAAGGAGACTTCGTGGCGTTCCTATTCGTCTTATGGAAGGCGCTAAACCTACCGGTGCCCACTAAGTGTCAGATTGACATGGCTAAGGTGCTGGCGAATGGAGACAACAAGAAGTTCATCTTACAGGCTTTCCGGGGTATCGGTAGGT    >  1:295457/1‑301`  
                                                                                                                                                                                                                                                                                                                  `GCTAAACGTATTCTCGCAGTGACCCAGAAGGGTGACGCATGGATTCGTGCGTTACAGGATACTATTCGTGAACTGCAACGTAAGTAGGAAATCAAGTAAGGAGGCAATGTGTCTACTCAATCCAATCGTAATGCGCTCGTAGTGGCGCAACTGAAAGGAGACTTCGTGGCGTTCCTATTCGTCTTATGGAAGGCGCTAAACCTACCGGTGCCCACTAAGTGTCAGATTGACATGGCTAAGGTGCTGGCGAATGGAGACAACAAGAAGTTCATCTTACAGGCTTTCAGTGGTATCGGTAAGg    >  1:253975/1‑300`  
                                                                                                                                                                                                                                                                                                                  `GCTAAACGTATTCTCGCAGTGACCCAGAAGGGTGACGCATGGATTCGTGCGTTACAGGATACTATTCGTGAACTGCAACGTAAGTAGGAAATCAAGTAAGGAGGCAATGAGTCTACTCAATCCAATCGTAATGCGCTCGTAGTGGCGCAACTGAAAGGATACTTCGTGGCGTTCCTATTCGTCTTATGGAAGGCGCTAAACCTACCGGTGCCCACTAAGTGTCAGATTGACATGGCTAAGGTGCTGTCAAATGGAGACAACAAGAAGTTCTTCTTAAAGGCTTTCCtgtgtatctgtctct    >  1:309879/1‑286`  
                                                                                                                                                                                                                                                                                                                  `GCTAAACGTATTCTCGCAGTGACCCAGAAGGGTGACGCATGGATTCGTGCGTTACAGGATACCATTCGTGAACTGCAACGTAAGTAGGAAATCAAGTCAGGAGGCAATGTGTCTACTCAATCCAATCGTAATGCGCTCGTAGCGGCGCAACTGAAAGGAGACTTCGTGGAGTTCCTATTCGTCTTATGGACGGCGCTAAACCTACCGGTGCCCACTAAGTGTCAGATTGACATGGCTAAGGTGCTGGCGAATGGGTACAACAAGAAGTTCATCTTACAGGCTTTCCGTGGTATCGGTAAGT    >  1:72606/1‑301`  
                                                                                                                                                                                                                                                                                                                  `GCTAAACGTATTCTCGCAGTGACCCAGAAGGGTGACGCATGGATTCGTGCATTACAGGATACCATTCGTGAACTGCAACGTTAGCAGGAAATCAAGTAAGGAGGCAATGTGTCTACTCAATCCAATCGTAATGCGCTCGTAGTGGCGCAACTGAAAGGAGACGTCGTGGCGTTCCTATTCGTCTTATGGAAGGCGCTAAACCTACCGGTGCCCACTAAGTCTCAGATTGACATGGCTAAGGTGCTGGCGAATGGAGACAACAAGAAGTCCATCTTACAGCATTTCCGTGGTATCGGTAAGT    >  1:81897/1‑301`  
                                                                                                                                                                                                                                                                                                                    `TAAACGTATGCTCGCAGTGACCCAGAAGGGTGACGCATAGATTCGTGCGTTACAGGATACTATTCGTGAACTGCAACGTAAGTAGGAAATCAAGTAAGGAGGCAATGTGTCTACTCAATCCAATCGTAATGCGCTCGTAGTGGCTCAACTGAAAGGAGACTTCGTGGCGTTCCTATTCGTCTTATGGAAGGCGCTAAACCTACCGGTGCCCACTAAGTGTCAGATTGACATGGCTAAGGTGCTGGCGAATGGAGACAACAAGAAGTTCATCTTACAGGCTTTCCGTGGTATCGGTAAGTCG  <  1:402385/301‑1`  

                                                                                                                                                                                                                                                                                                                                                                                                                                                                                                                                                                                                                                   
`ATTACATTCTTAGAGATGTTGGACACTGCGATGGCTCAGCGTATGCTTGCGGACCTTTCGGACCATGAGCGTCGCTCTCCGCAACTCTATAATGCTATTAACAAACTGTTAGACCGCCACAAGTTCCAGATTGGTAAGTTGCAGCCGGATGTTCACATCTTAGGTGGCCTTGCTGGTGCTCTTGAAGAGTACAAAGAGAAAGTCGGTGATAACGGTCTTACGGATGATGATATTTACACATTACAGTGATATACTCAAGGCCACTACAGATAGTGGTCTTTATGGATGTCATTGTCTATACGAGATGCT‑‑‑‑‑‑‑‑‑‑‑‑‑‑‑‑‑‑‑‑‑‑‑‑‑‑‑‑‑‑‑‑‑‑‑‑‑‑‑‑‑‑‑‑‑‑‑‑‑‑‑‑‑‑‑‑‑‑‑‑‑‑‑‑‑‑‑‑‑‑‑‑‑‑‑‑‑‑‑‑‑‑‑‑‑‑‑‑‑‑‑‑‑‑‑‑‑‑‑‑‑‑‑‑‑‑‑‑‑‑‑‑‑‑‑‑‑‑‑‑‑‑‑‑‑‑‑‑‑‑‑‑‑‑‑‑‑‑‑‑‑‑‑‑‑‑‑‑‑‑‑‑‑‑‑‑‑‑‑‑‑‑‑‑‑‑‑‑‑‑‑‑‑‑‑‑‑‑‑‑‑‑‑‑‑‑‑‑‑‑‑‑‑‑‑‑‑‑‑‑‑‑‑‑‑‑‑‑‑‑‑‑‑‑‑‑‑‑‑‑‑‑‑‑‑‑‑‑‑‑‑‑‑‑‑‑‑‑‑‑‑‑‑‑‑‑‑‑‑‑‑‑‑‑‑‑‑‑‑‑‑‑‑‑‑‑‑‑‑‑‑‑‑‑‑‑‑‑‑‑‑‑‑‑‑‑‑‑‑‑‑‑‑‑‑‑‑‑‑‑  >  NC_001604.1/36574‑36882`  
`‑‑‑‑‑‑‑‑‑‑‑‑‑‑‑‑‑‑‑‑‑‑‑‑‑‑‑‑‑‑‑‑‑‑‑‑‑‑‑‑‑‑‑‑‑‑‑‑‑‑‑‑‑‑‑‑‑‑‑‑‑‑‑‑‑‑‑‑‑‑‑‑‑‑‑‑‑‑‑‑‑‑‑‑‑‑‑‑‑‑‑‑‑‑‑‑‑‑‑‑‑‑‑‑‑‑‑‑‑‑‑‑‑‑‑‑‑‑‑‑‑‑‑‑‑‑‑‑‑‑‑‑‑‑‑‑‑‑‑‑‑‑‑‑‑‑‑‑‑‑‑‑‑‑‑‑‑‑‑‑‑‑‑‑‑‑‑‑‑‑‑‑‑‑‑‑‑‑‑‑‑‑‑‑‑‑‑‑‑‑‑‑‑‑‑‑‑‑‑‑‑‑‑‑‑‑‑‑‑‑‑‑‑‑‑‑‑‑‑‑‑‑‑‑‑‑‑‑‑‑‑‑‑‑‑‑‑‑‑‑‑‑‑‑‑‑‑‑‑‑‑‑‑‑‑‑‑‑‑‑‑‑‑‑‑‑‑‑‑‑‑‑‑‑‑‑‑‑‑‑‑‑‑‑‑‑‑‑‑‑‑‑‑‑‑‑‑‑‑‑cgagatgctAAACGTATTCTCGCAGTGACCCAGAAGGGTGACGCATGGATTCGTGCGTTACAGGATACTATTCGTGAACTGCAACGTAAGTAGGAAATCAAGTAAGGAGGCAATGTGTCTACTCAATCCAATCGTAATGCGCTCGTAGTGGCGCAACTGAAAGGAGACTTCGTGGCGTTCCTATTCGTCTTATGGAAGGCGCTAAACCTACCGGTGCCCACTAAGTGTCAGATTGACATGGCTAAGGTGCTGGCGAATGGAGACAACAAGAAGTTCATCTTACAGGCTTTCCGTGGTATCGGTAAGTCG  >  NC_001604.1/37265‑37564`

  

Alignment Legend

Aligned base mismatch/match (shaded by quality score): `ATCG/ATCG` `< 3 ≤` `ATCG/ATCG` `< 10 ≤` `ATCG/ATCG` `< 19 ≤` `ATCG/ATCG` `< 34 ≤` `ATCG/ATCG` `< 38 ≤` `ATCG/ATCG`

Unaligned base: `atcg`    Masked matching base: `atcg`    Alignment gap: `‑`    Deleted base: `‑`

Reads not counted as support for junction

read\_name Not counted due to insufficient overlap past the breakpoint.

read\_name Not counted due to not crossing MOB target site duplication.
